# Supplementary material for: Systematic Identification of circRNAs in Alzheimer’s Disease
Source: Genes (Basel). 2021 Aug 18;12(8):1258. doi: 10.3390/genes12081258 (PMC8391980; doi:10.3390/genes12081258)

LEGENDS OF SUPPLEMENTAL FIGURES

Figure S1. Aligned vs unaligned identified reads. (A) Graph shows the average number of aligned and unaligned reads per study in millions. Aligned sequences were generated using the STAR aligner which had a successful mapping rate of 70-90%. **(B)** The number of identified circRNA in each study using CIRCexplorer2's *parse* and *annotate* modules. All circRNAs were identified via their unique backspliced junction sequence.

Figure S2. Common differentially expressed circRNA across brain studies and plasma studies. (A) An upset plot (see **Figure 3** legend) showing the differentially expressed (re: expression levels significantly increased or decreased) circRNA that were common across the four brain studies. This plot does account for circRNAs that would be upregulated in one study and downregulated in another. **(B)** A Venn diagram depicting differentially expressed circRNAs that were common across the two plasma studies.

LEGENDS OF SUPPLEMENTAL TABLE

Supplemental Table S1. CircRNAs from Figure 3. The circRNAs more highly abundant in AD, as well as those less abundant in AD relative to normal brain study groups are listed. Each study is listed in a separate spreadsheet.

Supplemental Table S2. CircRNAs from figure 5A. The common differentially abundant circRNAs between PRJNA574438 or GSE161199 individually, and at least 3 of the 4 brain studies. The 53 gene loci (49+4) correspond to 83 (79+4) individual circRNAs.

Supplemental Table S3. CircRNAs from Figure 5B. CircRNAs that were found to be differentially abundant in at least one plasma study and at least one any brain study.

Cochran et al., Figure S1

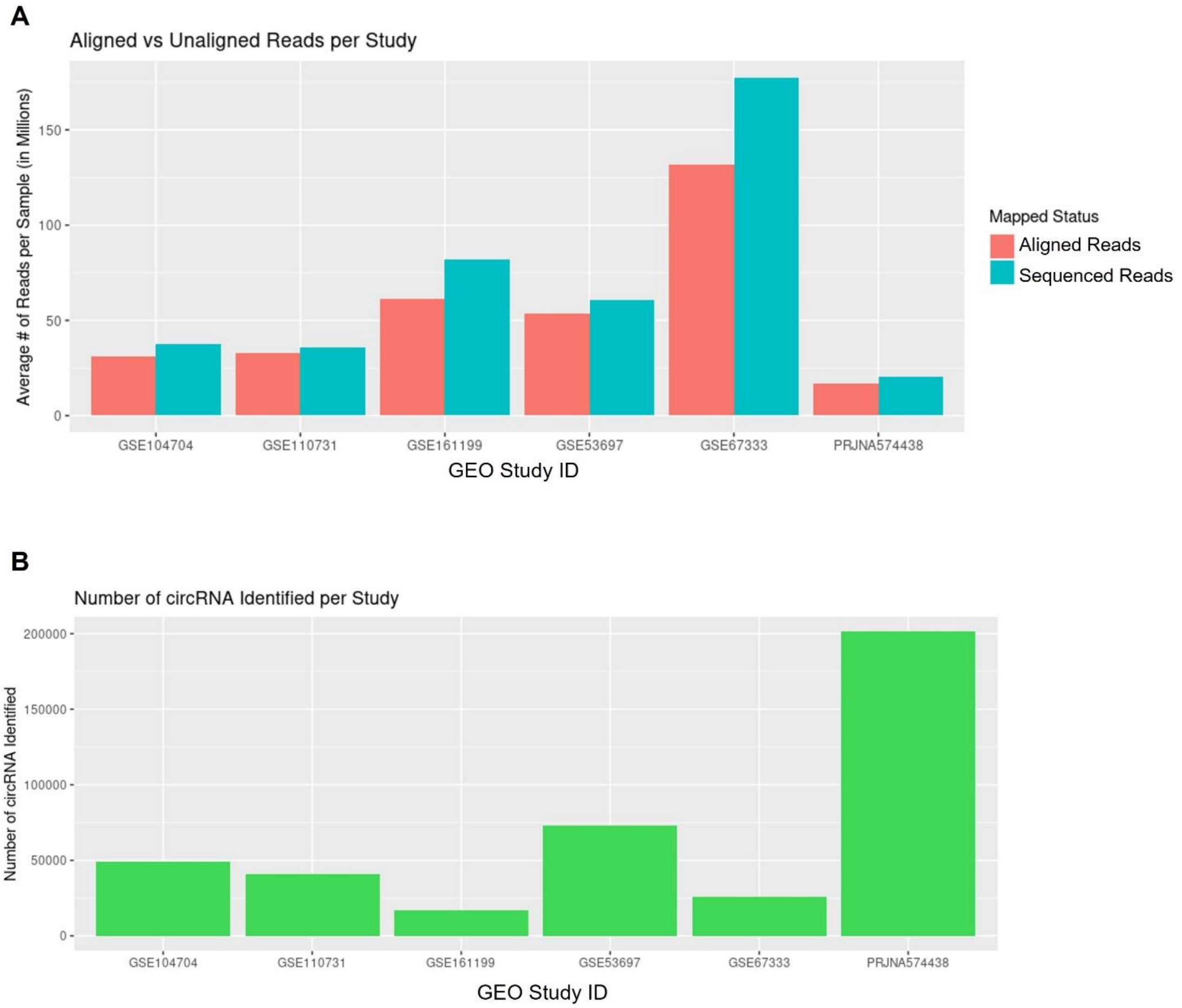

Cochran et al., Figure S2

A

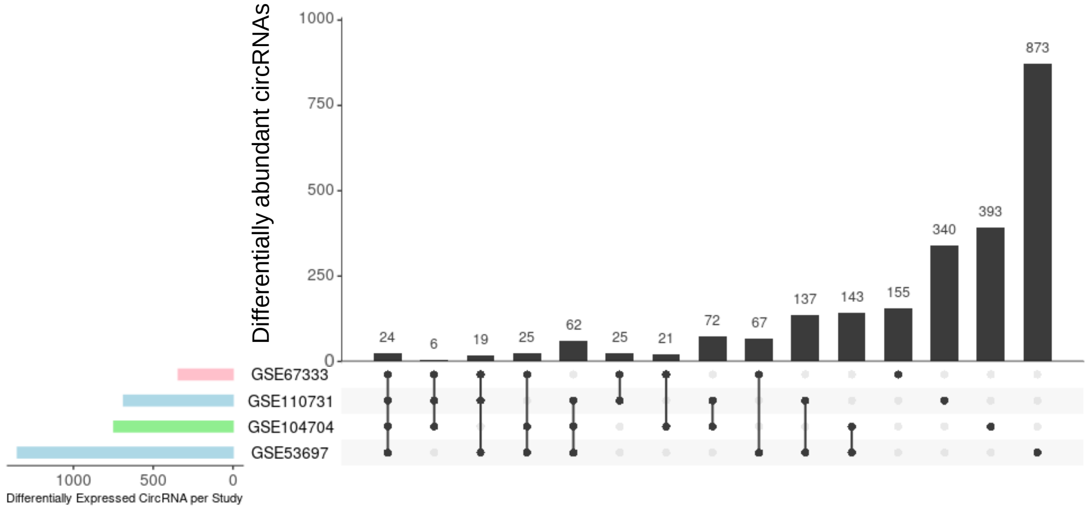

B

Differentially abundant circRNAs

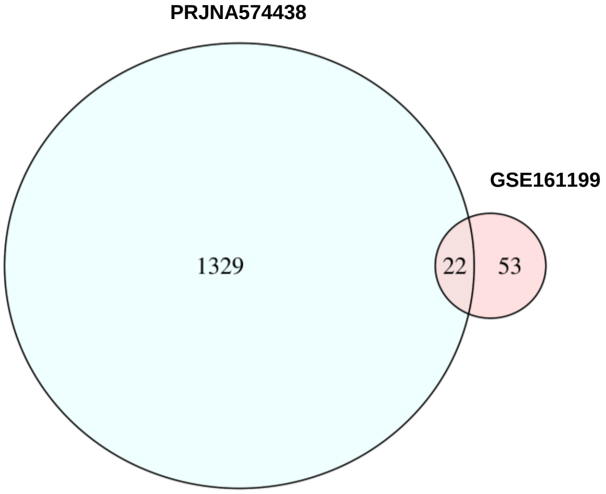

Supplement: Supplementary file 1 [file genes-12-01258-s001.zip › Cochran et al. supplemental text and figures final.pdf]
